# Myocardial citrullination in rheumatoid arthritis: a correlative histopathologic study

**DOI:** 10.1186/ar3752

**Published:** 2012-02-24

**Authors:** Jon T Giles, Justyna Fert-Bober, Jin Kyun Park, Clifton O Bingham, Felipe Andrade, Karen Fox-Talbot, Dimitrios Pappas, Antony Rosen, Jennifer van Eyk, Joan M Bathon, Marc K Halushka

**Affiliations:** 1 Division of Rheumatology, Columbia University, College of Physicians & Surgeons, 630 W 168th St, New York, NY 10032 USA; 2 Division of Cardiology, The Johns Hopkins University School of Medicine, The Johns Hopkins University, 600 N Wolfe St, Baltimore, MD 21287, USA; 3 Division of Rheumatology, The Johns Hopkins University School of Medicine, The Johns Hopkins University, 600 N Wolfe St, Baltimore, MD 21287, USA; 4 Department of Pathology, The Johns Hopkins University School of Medicine, The Johns Hopkins University, 600 N Wolfe St, Baltimore, MD 21287, USA

## Abstract

**Introduction:**

The aim of this study was to explore the presence and localization of myocardial citrullination in samples from rheumatoid arthritis (RA) patients compared to rheumatic and non-rheumatic disease control groups.

**Methods:**

Archived myocardial samples obtained during autopsy from 1995 to 2009 were assembled into four groups: RA; scleroderma; fatal myocarditis; and non-rheumatic disease controls. Samples were examined by immunohistochemistry (IHC) for the presence and localization of citrullination and peptidyl arginine deiminase enzymes (PADs) by a single cardiovascular pathologist blinded to disease group and clinical characteristics.

**Results:**

Myocardial samples from seventeen RA patients were compared with those from fourteen controls, five fatal myocarditis patients, and ten scleroderma patients. Strong citrullination staining was detected exclusively in the myocardial interstitium in each of the groups. However, average and peak anti-citrulline staining was 59% and 44% higher, respectively, for the RA group compared to the combined non-RA groups (*P *< 0.05 for both comparisons). Myocardial fibrosis did not differ between the groups. In contrast to citrullination, PADs 1 to 3 and 6 were detected in cardiomyocytes (primarily PADs 1 and 3), resident inflammatory cells (primarily PADs 2 and 4), and, to a smaller extent, in endothelial cells and vascular smooth muscle cells. PAD staining did not co-localize with anti-citrulline staining in the interstitium and did not vary by disease state.

**Conclusions:**

Staining for citrullination was higher in the myocardial interstitium of RA compared to other disease states, a finding that could link autoimmunity to the known increase in myocardial dysfunction and heart failure in RA.

## Background

Rates of heart failure and diastolic dysfunction are increased in rheumatoid arthritis (RA) patients compared to non-RA controls independent of coronary artery disease [1], suggesting that myocardial remodeling occurs as part of the RA disease process. The phenotype of heart failure in RA differs from that of non-RA patients, characterized by fewer symptoms, lower blood pressure, and higher ejection fraction at presentation [2], suggesting that the pathophysiologic mechanisms underlying the progression to heart failure in RA may differ from the general population. Reports of increased myocarditis and vasculitis in autopsied RA hearts compared to controls date back more than five decades [3,4]. However, the myocardium as a potential autoimmune target in rheumatoid arthritis has received little direct investigation since that time.

Recently, we reported an association of a higher concentration of serum anti-cyclic citrullinated peptide (anti-CCP) antibodies with lower myocardial mass and smaller left ventricular chamber volumes in RA patients without known cardiovascular disease [5]; raising the possibility that RA-specific autoimmunity against citrullinated proteins might mediate changes to myocardial morphology that, in turn, may affect myocardial function. Citrullination, the post-translational modification of arginine to citrulline catalyzed by a set of peptidyl-arginine deiminase enzymes (PADs), is abundant in the rheumatoid synovium but not restricted to RA [6]. Citrullinated proteins have been identified in the affected tissues of a number of inflammatory conditions, such as muscle in myositis patients, myelin sheaths in multiple sclerosis, and intestinal mucosa in inflammatory bowel disease [7] and in non-pathologic tissues with homeostatic functions, such as keratinized epithelium.

What are the potential links between circulating anti-citrullinated peptide antibodies (ACPA) and the myocardium? Many of the protein targets that undergo citrullination in rheumatoid synovium (that is, vimentin, enolase, fibronectin) and are targets for ACPA in RA [8] are also present in the myocardium [9]. However, it is unknown whether citrullination of myocardial proteins is present in RA. For this investigation, we explored the presence, abundance, and localization of citrullination in archived myocardial samples from RA patients compared to a variety of rheumatic and non-rheumatic disease control groups. Further, we explored co-localization of citrullination with histologic features and the presence of human PAD isotypes. We hypothesized that myocardial citrullination would be unique to, or more abundant in, RA compared to other conditions, and that myocardial regions demonstrating citrullination would co-localize with evidence of tissue damage (for example, myocarditis, fibrosis) and human PAD expression.

## Methods

### Myocardial sample identification

#### RA sample identification

The study was approved by the Institutional Review Board of The Johns Hopkins Hospital, which waived the need for obtaining decedent informed consent for use of archived myocardial samples and records. Myocardial samples acquired at autopsy from individuals with RA were identified using a series of ascertainment and adjudication steps. First, an initial search was conducted of the Johns Hopkins Anatomic Pathology database for mention of the term 'rheumatoid arthritis' within the text of any autopsy report performed between January 1, 1995 and July 1, 2009. The start date was chosen as RA diagnosis and treatment practices would be more consistent with current practices, and preservation and organization of samples would be superior to those stored prior. Also, case adjudication was facilitated using the Electronic Patient Record system of The Johns Hopkins Hospital, with records available from 1995 onward. We created a standard data abstraction tool for each disease group and determined, *a priori*, the extractable data elements that would constitute definite, possible, and unlikely for each condition. Categorization as RA required at least two outpatient clinic evaluations by a rheumatologist in which RA was identified as the diagnosis with indication of active small joint polyarthritis or clinical joint deformities typical for RA plus treatment with medications typically used for the treatment of RA (that is, biologic and non-biologic disease modifying anti-rheumatic drugs [DMARDS]). Possible and unlikely cases had more limited clinical information available. Data were abstracted by a single study team member (JP and adjudicated by agreement between two study rheumatologists (JTG and JMB). Of the 49 potential cases with 'RA' mentioned in the autopsy report, 20 adjudicated RA cases, five possible, and 24 unlikely cases were identified. Of the RA cases, 17 had available myocardial samples and constituted the RA group used for histopathology.

#### Control sample identification

Controls without rheumatic disease or myocarditis were selected in a similar manner as described for the RA cases and group matched on age to the RA group. For the scleroderma group, an initial Pathology database query identified 28 potential cases with specimens since 1995. Of these, further review with the scleroderma data abstraction tool yielded 14 adjudicated definite, three possible, and 11 unlikely cases. Among the definite cases, 10 had available myocardial samples. For myocarditis, only lymphocytic and eosinophilic myocarditis cases were included. Of the 56 potential cases identified from the initial Pathology database query, 14 met inclusion criteria for the desired myocarditis characteristics; however, only five had myocardial specimens available for histopathology.

### Clinical characteristics

Gender and ethnicity were collected from medical records. Age at death, postmortem interval ([PMI], the time in hours between death and autopsy), and the presence and severity of coronary atherosclerosis were collected from autopsy records. Other characteristics, including cardiac history, were not uniformly available or reliable among all individuals with samples, and were thus not used for analysis beyond confirmation of diagnosis.

### Histopathology and immunohistochemistry

Autopsy blocks, of the left ventricular free wall, were obtained from the archives of the Johns Hopkins Department of Pathology. Five-micron sections were cut and placed on charged slides. These were immersed in Trilogy rinse (Cell Marque, Hot Spring, AR, USA) and placed in an electric pressure cooker until reaching 127°C and 17 psi. Slides stained for citrulline were modified in a strong acid solution containing 2.3-butanedoine for 3 h at 37°C. Endogenous peroxidase activity was blocked by incubation in 0.3% H_2_O_2 _in methanol for 18 min. Non-specific protein activity was blocked by incubation with a non-serum protein solution (DAKO Corporation, Carpinteria, CA, USA). Slides were incubated with antibodies targeting PAD1 (1:1000 Abcam, Cambridge, MA, USA), PAD2 (1:200, Abcam), PAD3 (1:500, Abcam, PAD4 (1:150, Sigma-Aldrich, St Louis, MO, USA), PAD6 (1:150 Abcam) or citrulline (1:1000, Millipore Corporation, Billerica, MA) overnight at 4°C. For anti-PAD1, slides underwent an additional treatment with HRP polymer detection kit (Invitrogen, Carlsbad, CA, USA). Sections were then incubated with biotinylated donkey anti-rabbit IgG (Jackson ImmunoResearch Laboratories, West Grove, PA, USA).

Immunoperoxidase staining was performed using Vectastain ABC Elite (Vector Labs, Burlingame, CA, USA). The avidin-biotin complex was visualized using 3.3' diaminobenzidine (DAB) peroxidase substrate (Vector Labs). Sections were counterstained in hematoxylin (Richard-Allen, Kalamazoo, MI, USA).

All cardiac tissue scoring of the myocardium was performed by a single-blinded cardiovascular pathologist (MKH). Citrullination staining was based on a 5-point scale (1 to 3 in 0.5 increments) corresponding to minimal, moderate, and marked citrullination staining. Both an overall staining score and a peak staining score were generated for each tissue as there was some heterogeneity in staining intensity across the slides. Cardiac hypertrophy was measured subjectively based upon the surrogate nuclear features of size, hyperchromasia, and irregularity of the nuclear membranes [10]. Myocarditis was initially reported in the autopsy record and was confirmed through the histopathologic findings of lymphocytic infiltration causing myocyte injury. Areas of fibrosis included both interstitial (fibrosis between myocytes) and replacement (fibrotic bands replacing myocytes) types and were scored using the same semi-quantitative scale. Scoring of PAD staining was also performed blinded using the same 1 to 3 scale. Staining was independently evaluated and scored for the myocytes, smooth muscle cells, endothelium, and leukocytes.

### Statistical analysis

The distributions of clinical and histologic characteristics were examined and compared between groups using analysis of variance (ANOVA), with Bonferonni correction, for normally distributed continuous variables, the Kruskal-Wallis test for non-normally distributed continuous variables, and the chi-square goodness of fit test or Fisher's exact test, as appropriate, for categorical variables. Generalized linear models were constructed to explore the associations of patient characteristics with average and peak citrullination scores in the RA and control groups, with heterogeneity in the associations between groups tested using analysis of co-variance (ANCOVA). The Shapiro-Wilk test was used to ensure normality of the modeled outcome variables across the extent of independent variables. All calculations were performed using Intercooled Stata 10 (StataCorp, College Station, TX, USA). In all tests, a two-tailed α of 0.05 was utilized.

## Results

Characteristics of the disease groups are summarized in Table 1. Compared to the control group, the RA group did not differ in magnitude or significance by age at autopsy, gender, ethnicity, PMI, or the presence/extent of reported coronary atherosclerosis. In addition, there were no differences between the RA and control groups in histologic evidence of myocarditis, myocardial interstitial fibrosis, replacement fibrosis, or cardiomyocyte hypertrophy. For the myocarditis and scleroderma groups, there were differences in demographics compared with the RA and control groups. Specifically, the scleroderma group was significantly younger at death, on average, than either the RA or control groups. The proportion of Caucasians was lower in the scleroderma group compared to the RA or control groups, although these differences did not reach statistical significance. Gender was similar among the groups, except for the myocarditis group, in which the proportion of women was lower than either the RA or control groups (*P *= 0.028 and 0.26, respectively). PMI did not differ significantly for either the myocarditis or scleroderma groups compared to the RA or control groups.

**Table 1:** Demographic and histologic characteristics according to study group

	RA (*n *= 17)	Control (*n *= 14)	Myocarditis (*n *= 5)	Scleroderma (*n *= 10)	*P* ^a^
*Demographics and reported autopsy features*					
Age, years	68 ± 12	67 ± 12	61 ± 13	54 ± 13	0.85
Female, *n *(%)	15 (88)	11 (79)	2 (40)	7 (70)	0.64
Caucasian, *n *(%)	12 (71)	12 (86)	4 (80)	5 (50)	0.41
PMI, median (%)	19 (13-23)	20 (9-28)	25 (21-27)	24 (19-39)	0.92
Coronary atherosclerosis, any reported; *n *(%)	7 (41)	7 (50)	2 (40)	4 (40)	0.62
Mild or moderate, *n *(%)	4 (24)	6 (43)	1 (20)	3 (30)	0.44
Severe, *n *(%)	3 (18)	1 (7)	1 (20)	1 (10)	0.60
*Histologic findings*					
Myocarditis, *n *(%)	0 (0)	1 (6)	5 (100)	0 (0)	1.00
Interstitial fibrosis, *n *(%)	14 (81)	12 (86)	3 (60)	7 (70)	1.00
Replacement fibrosis, *n *(%)	3 (19)	4 (29)	1 (20)	3 (30)	0.68
Cardiomyocyte hypertrophy, *n *(%)	11 (63)	9 (64)	2 (40)	7 (70)	1.00

^a^*P *value for RA *vs*. control groups.

PMI, postmortem interval; RA, rheumatoid arthritis.

### Quantification and localization of myocardial citrullination

**Figure 1:**
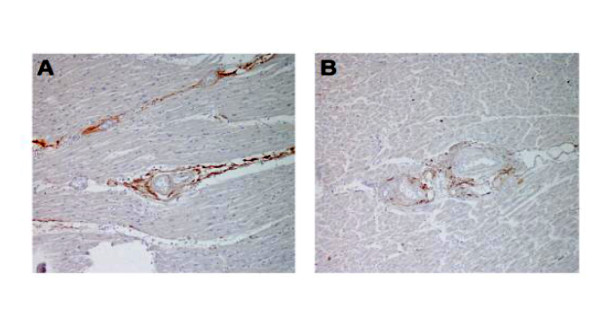
**Representative sections are shown demonstrating anti-citrulline staining in rheumatoid arthritis myocardium (A) and control myocardium (B)**. Citrullination was restricted to the myocardial interstitium in both groups, with staining intensity qualitatively higher in the RA group. Interstitial citrullination was also observed in myocardial samples from fatal myocarditis and scleroderma patients with a qualitative intensity similar to the control group (not depicted).

**Figure 2:**
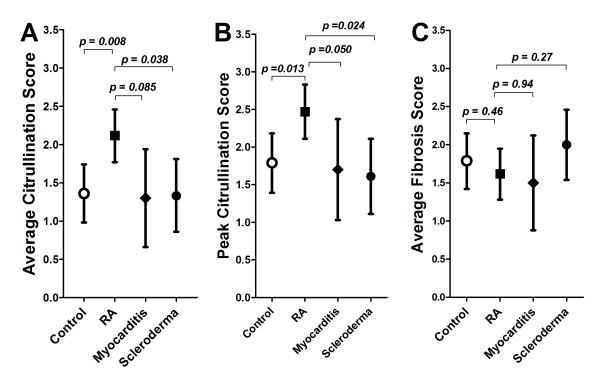
**Mean average **(A) **and peak **(B) **citrullination scores, and mean average fibrosis score **(C) **according to disease group**. Mean and 95% confidence intervals are depicted.

Histologic staining for citrullination was localized exclusively in the perivascular interstitium of RA (Figure 1a), control (Figure 1b), myocarditis, and scleroderma samples. At an antibody concentration of 1:1000, citrullination was not identified in the cardiomyocytes, endothelium, vascular smooth muscle cells (VSMCs), or infiltrating myocardial inflammatory cells. The mean average (Figure 2a) and peak (Figure 2b) citrullination scores were similar between the control, myocarditis, and scleroderma groups. Compared to these groups, the mean average and peak myocardial anti-citrulline staining intensity scores were 59% and 44% higher, respectively, for the RA group compared to the combined non-RA groups (*P *< 0.001 for both differences). These differences remained significant even after adjusting for age, gender, and ethnicity. In contrast, mean average scores for staining for myocardial fibrosis were similar between all groups (Figure 2c). Areas of high intensity citrullination staining did not co-localize with areas of inflammation seen in myocarditis.

### Associations of myocardial citrullination with patient characteristics

**Figure 3:**
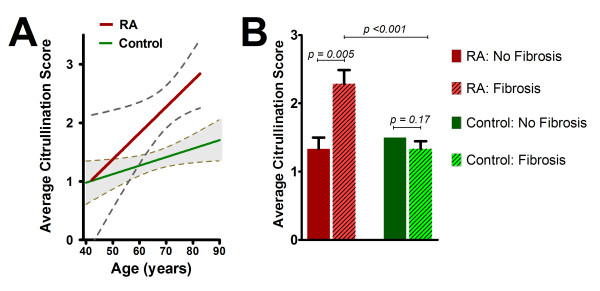
**Associations of age **(A) **and myocardial fibrosis **(B) **with average citrullination, according to RA status**. For age, the average linear association is depicted (least-squares estimate) for the RA (β = 0.044; *P *< 0.05) and control (β = 0.032; *P *< 0.05), a difference in slopes that was significant (*P *value for interaction = 0.040). Dashed lines in panel A indicate 95% confidence intervals. For panel B, bars indicate means with fences representing the standard error of the mean.

We explored the associations of the uniformly available demographic and histologic characteristics (that is, age, gender, ethnicity, PMI, and histologic evidence of myocarditis, interstitial and replacement fibrosis, and cardiomyocyte hypertrophy) with mean average and peak citrullination scores. Higher age was associated with higher mean average citrullination in both the RA and control groups (Figure 3a); however, the magnitude of the association of age with average citrullination was greater in the RA *vs*. control group, and was only statistically significant in the RA group. Gender, ethnicity, PMI, and presence/extent of reported coronary atherosclerosis were not associated with the extent of citrullination in either the RA or control groups (data not shown). Among histologic findings, the mean average citrullination score was almost 1 unit higher among RA patients with evidence of interstitial fibrosis compared to those without fibrosis (Figure 3b; *P *= 0.005). In contrast, an association of interstitial fibrosis with citrullination was not observed among controls. The magnitude, direction, and significance of the associations of age and interstitial fibrosis with average citrullination score were not substantially changed when age and interstitial fibrosis were co-modeled. Cardiomyocyte hypertrophy was not associated with citrullination in either group. There was no significant association of myocarditis with citrullination in the control group and, since no RA patients had evidence of myocarditis, no association could be explored in this group. Associations of patient characteristics with mean peak citrullination scores were similar to those observed with average citrullination in magnitude and direction among both groups (data not shown).

### Quantification and localization of myocardial PADs

**Figure 4:**
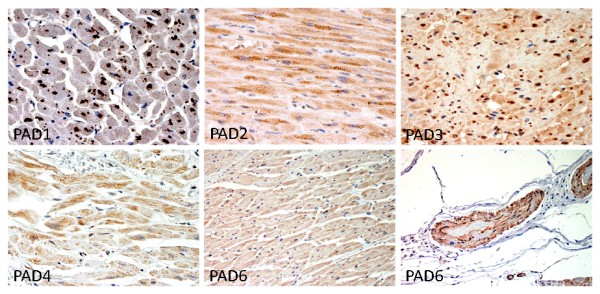
**Representative images of PAD staining of the cardiac myocytes**. The second PAD6 image demonstrates vascular smooth muscle cell and endothelial cell staining (original magnifications 100×).

Semi-quantitative assessments and localization of PADs from myocardial samples are summarized in Table 2. There were no meaningful differences in PAD staining intensity in any cellular compartment between RA and control samples. In contrast to citrullination, PADs were not detected in the extracellular matrix of the myocardial interstitium and localization of PAD isotypes to specific regions of the myocardium was noted. PAD1 strongly stained perinuclear cytoplasmic granules of cardiomyocytes (Figure 4, top left) and, to a much lesser extent, leukocytes. PAD2 was observed primarily in leukocytes (Figure 4, top middle) and at very low background levels among the other myocardial cell types. Strong PAD3 staining was observed in cardiomyocytes (Figure 4, top right), which, in contrast to the cytoplasmic granules staining for PAD1, primarily stained in the region of the nucleus. Weaker PAD3 staining was observed in vascular smooth muscle cells (VSMCs) and in leukocytes. Strong PAD4 staining was observed in leukocytes (Figure 4, bottom left) and weak staining was observed in other cell types. PAD6 was observed in low background levels diffusely in the cytoplasm of cardiomyocytes (Figure 4, bottom middle), but moderately stained VSMCs and endothelium (Figure 4, bottom right).

**Table 2:** Summary of the presence, extent, and localization of citrullination and PAD isotypes in the myocardium of RA patients

	Cardiomycocytes	VSMCs	Endothelium	Leukocytes	Extracellular matrix
Citrullination	Absent	Absent	Absent	Absent	+++
PAD1	+++^a^	Absent	Absent	+	Absent
PAD2	+^b^	+	+	+++	Absent
PAD3	+++^a, c^	++	+	++	Absent
PAD4	+^b^	+	+	+++	Absent
PAD6	+^b^	++	++	Absent	Absent

^a^Granular staining pattern; ^b^cytoplasmic staining pattern; ^c^nuclear staining pattern.

PAD, peptidyl-arginine deiminase; VSMC, vascular smooth muscle cells.

## Discussion

To the best of our knowledge, this is the first report of the presence of myocardial citrullination in any disease state, and the first histologic description of staining patterns of all known human PAD isotypes in myocardial tissues. These data provide evidence that: (1) citrullinated proteins are present in the myocardial interstitium of diverse disease states, but significantly increased in RA; (2) demographics, postmortem interval, and the presence or extent of reported coronary atherosclerosis did not account for higher citrullination in RA; and (3) a variety of PADs are present in the myocardium, but they do not co-localize with interstitial citrullination. Our finding that citrullination was indeed present in the myocardium extends the list of tissues known to undergo this form of post-translational modification. Although we did not identify specific citrullinated proteins in this investigation, the localization of citrullination to the interstitium may provide clues as to likely candidates. Proteins found in the interstitium are primarily those of extracellular matrix (ECM) and proteins that interact with ECM (for example, collagens, fibronectin, laminin) [11]; however, while these are the most common interstitial proteins, the list of additional candidate proteins remains extensive. Our finding that age was more strongly associated with citrullination in RA compared to controls could suggest that citrullination is an expected aging-related phenomenon accelerated in RA by processes that remain to be elucidated, perhaps related to accelerated apoptosis or autophagic death of cardiomyocytes or cardiac leukocytes. Neither myocarditis nor myocyte necrosis were frequent among RA samples, suggesting that fulminant processes such as these may not be the triggers for citrullination. Moreover, the lack of citrullination above control levels among fatal myocarditis patients further supports a lack of association. Another interesting finding was higher citrullination scores among RA samples with evidence of interstitial fibrosis. The association suggests that the two processes may be linked; however, whether interstitial fibrosis and citrullination are causally linked, or merely epiphenomena, is not evident from our study.

There is precedent for other mechanisms of post-translational modification of myocardial proteins leading to changes in cardiac structure and function. Phosphorylation, oxidation, and glycation of sarcomeric proteins cause morphologic changes to proteins that lead to increased protein cross-linking, resulting in increased ventricular stiffness [12]. Beyond direct structural effects related to altered protein-protein interaction, accumulation of modified proteins has been shown to mediate myocardial fibrosis via modulation of the expression of proteases and transcription factors in myocardial fibroblasts [13]. It is unknown whether citrullination of myocardial proteins leads to altered interaction of myocardial proteins or cellular processes leading to interstitial fibrosis similar to other post-translational modifications; however, our findings warrant additional study into possible mechanisms.

Although citrullination appears to be a generalized process, autoantibodies targeting citrullinated proteins are relatively specific for RA and, although occasionally observed in other autoimmune conditions, are uncommonly observed in healthy individuals [14]. Thus, RA patients may be uniquely at risk for autoreactivity against proteins that, upon citrullination, function as neo-epitopes for anti-citrulline autoantibodies. RA patients are known to exhibit a broader repertoire of reactivity against citrullinated peptides compared to non-RA patients with ACPA antibodies [8], a process potentiated by epitope spreading [15]. Although it is not known from our study whether myocardial citrullinated proteins are immune targets for circulating autoantibodies, or whether autoimmunity against citrullinated proteins mediates phenotypic modifications to cardiac structure or function, this is a goal of ongoing investigation.

The cellular localization of PAD isotypes within the myocardium has not been previously described. While PADs 1 and 3 are primarily regarded as epidermal in origin, mRNA for PAD1 has been identified in muscle tissue [16]. In addition, our finding of PADs 2 and 4 primarily in leukocytes is expected from previous investigation [17]. Somewhat unexpected was the observation of a clear separation between the locations of citrullination and PADs in the myocardium in all of the disease groups, a finding that warrants further study.

Some notable study limitations deserve acknowledgment. Most notably, samples derived from autopsy may not represent processes active in life, findings may be influenced by cause of death, samples tend to be derived from older individuals and thus may misrepresent natural history over the course of disease, and histopathology may be affected by the interval between death and sample collection (that is, PMI). Because of slow progression and infrequency of fulminant symptoms, the diagnosis and management of myocardial dysfunction in RA patients rarely requires endomyocardial biopsy, limiting the spectrum of myocardial samples available. Even so, samples collected for clinical diagnostics could be biased by the factors necessitating biopsy (that is, skewing toward patients with extreme findings). Regarding the potential for PMI to confound our findings, we did not observe an influence of PMI on anti-citrulline detection, suggesting that citrullination is stable post mortem. Another limitation was the lack of clinical data, particularly for RA features and treatments. While richer clinical data would be helpful in exploring factors linked to citrullination within the RA group, notably the presence or absence of ACPA, the absence of the clinical characteristics does not impugn the differences detected comparing RA *vs*. non-RA disease states. As with any IHC-based study, antibody titer can affect where staining is observed. We used a low titer for our anti-modified citrulline antibody and saw robust staining in the extracellular matrix. A 10-fold higher titer of antibody did show myocardial staining, but it was difficult to discern this from background staining indicating that myocyte citrullination needs to be confirmed by a separate method. Finally, a strength of the investigation was the development *a priori *of standardized data abstraction tools and criteria for each disease group, with samples analyzed only for cases identified as definite or highly probable.

## Conclusions

In summary, our novel detection of citrullinated proteins within the interstitium of the myocardium may have implications for cardiac physiology, both for RA patients and the general population. More extensive citrullination linked to the RA disease state, and the more robust association of age and interstitial fibrosis observed in RA compared to non-RA groups, may contribute to higher rates of myocardial dysfunction and overt heart failure in RA that deserves additional investigation.

## Abbreviations

ACPA: anti-citrullinated peptide antibody; ANCOVA: analysis of covariance; ANOVA: analysis of variance; DAB: diaminobenzidine; DMARD: disease modifying anti-rheumatic drug; ECM: extra-cellular matrix; PAD: peptidyl-arginine deiminase; PMI: postmortem interval; RA: rheumatoid arthritis; VSMC: vascular smooth muscle cells.

## Competing interests

The authors declare that they have no competing interests.

## Authors' contributions

All authors contributed to the design and conception of the study, and were involved in the drafting of the manuscript. MKH and KFT performed the histopathology. JTG, JP, and LMB reviewed patient records and adjudicated cases. JTG performed the statistical analysis. All authors have read and approve the final manuscript.
